# Endothelin-1 in exhaled breath condensate of allergic asthma patients with exercise-induced bronchoconstriction

**DOI:** 10.1186/1465-9921-8-76

**Published:** 2007-10-31

**Authors:** Ziemowit Zietkowski, Roman Skiepko, Maria M Tomasiak, Anna Bodzenta-Lukaszyk

**Affiliations:** 1 Department of Allergology and Internal Medicine, Medical University of Bialystok, Poland

## Abstract

**Background:**

Exercise-induced bronchoconstriction (EIB) is a highly prevalent condition, whose pathophysiology is not well understood. Endothelins are proinflammatory, profibrotic, broncho- and vasoconstrictive peptides which play an important role in the development of airway inflammation and remodeling in asthma. The aim of the study was to evaluate the changes in endothelin-1 levels in exhaled breath condensate following intensive exercise in asthmatic patients.

**Methods:**

The study was conducted in a group of 19 asthmatic patients (11 with EIB, 8 without EIB) and 7 healthy volunteers. Changes induced by intensive exercise in the concentrations of endothelin-1 (ET-1) in exhaled breath condensate (EBC) during 24 hours after an exercise challenge test were determined. Moreover, the possible correlations of these measurements with the results of other tests commonly associated with asthma and with the changes of airway inflammation after exercise were observed.

**Results:**

In asthmatic patients with EIB a statistically significant increase in the concentration of ET-1 in EBC collected between 10 minutes and 6 hours after an exercise test was observed. The concentration of ET-1 had returned to its initial level 24 hours after exercise. No effects of the exercise test on changes in the concentrations of ET-1 in EBC in either asthmatic patients without EIB or healthy volunteers were observed. A statistically significant correlation between the maximum increase in ET-1 concentrations in EBC after exercise and either baseline F_ENO _and the increase in F_ENO _or BHR to histamine 24 hours after exercise in the groups of asthmatics with EIB was revealed.

**Conclusion:**

The release of ET-1 from bronchial epithelium through the influence of many inflammatory cells essential in asthma and interactions with other cytokines, may play an important role in increase of airway inflammation which was observed after postexercise bronchoconstriction in asthmatic patients.

## Background

The airway response to exercise in most asthmatic patients has been known as a postexercise fall in lung function followed by a spontaneous recovery. This classical response is labelled as exercise-induced bronchoconstriction (EIB). Despite the wide prevalence and clinical significance of EIB, the mechanisms responsible for it have yet to be clearly described [1]. Also the findings related to the participation of inflammatory mediators in either the maintenance or induction of bronchoconstriction provoked by exercise are conflicting [2].

Endothelins are proinflammatory, profibrotic, broncho- and vasoconstrictive peptides. Endothelin-1 (ET-1) has been demonstrated in the airway epithelial and endothelial cells and is involved in the pathogenesis of bronchial asthma. ET-1 accelerates DNA synthesis and cellular proliferation in human lung fibroblasts. It is also suggested that ET-1 influences asthmatic inflammation, provoking concentration and proliferation of bronchial smooth muscle cells and subepithelial fibrosis. This leads to airway remodeling and severe bronchial hyperreactivity [3]. Recent studies suggest the essential role of ET-1 in bronchoconstriction, mucus secrection, and plasma exudation [4-7].

In our previous reports, we suggest that during exercise-induced bronchoconstriction, changes in the function of the pulmonary endothelium occur [8]. Based on these findings, it is considered that the release of inflammatory mediators, such as endothelin-1, as well as adhesion molecules, through enhancing the migration of inflammatory cells as well as interactions with other cytokines essential in asthma, may contribute to the exacerbation of asthmatic inflammation in the airways and bronchial hyperreactivity after exercise.

The airway epithelium is involved in allergic inflammatory processes, producing and releasing endothelins, cytokines, chemokines, and growth factors, as well as eicosanoides active in the pathophysiology of airway diseases [9]. This study was designed to clarify the possible role of ET-1 released from bronchial epithelial cells in the pathogenesis of EIB, particular in the inflammatory basis of this condition. ET-1 levels were measured in exhaled breath condensate (EBC), collecting by cooling exhaled air – noninvasive procedure, easily performed and effort independent, a rapid method for obtaining samples from the lower respiratory tract [10].

The aim of the study was to evaluate the changes in ET-1 in EBC following intensive exercise in asthmatic patients and to establish the possible correlation of these measurements with the parameters of airway inflammation and their changes after exercise.

## Materials and methods

### Patients

The study was conducted on a group of 19 mild allergic asthma patients. Asthma was diagnosed according to the criteria recommended by the GINA 2002 [11]. All patients had been in a stable condition, free from acute exacerbations and respiratory tract infections for the previous two months. Patients with other factors which could change F_ENO _levels (except for asthma, features of atopy, or allergic rhinitis) were excluded. In all patients the tests were performed out of pollen season. Prior to the beginning of this study, patients were allowed to take short- and long-acting β_2_-agonists. Asthmatic patients who had been treated with drugs other than β_2_-agonists (inhaled steroids, antileucotrienes) in the past three months, were excluded from the study. F_ENO _measurement, skin prick tests with commonly encountered aeroallergens (house dust mites, trees, weeds, grasses, cat, Alternaria and Cladosporium), flow/volume spirometry, and a bronchial provocation test with histamine were performed on each asthmatic patient before qualifying for the exercise test.

Seven healthy volunteers were used as a negative control group. All of them underwent F_ENO_, flow/volume spirometry, and skin prick tests with common aeroallergens. They had FEV_1 _> 80% predicted. They were free of respiratory tract infection for 2 months prior to the study and from other significant illnesses known to affect F_ENO _measurements. Asthma patients and healthy volunteers were non-smokers and during the last year have not been passive smokers.

Total IgE and peripheral blood eosinophilia were determined in all asthmatic patients and healthy volunteers. In all asthmatic patients and healthy volunteers, an exercise test on the bicycle ergometer was performed.

24 hours after exercise, measurement of F_ENO _and a bronchial provocation test with histamine were performed.

The study protocol was approved by the Ethics of Research Committee of the Medical University of Bialystok, agreement number: R-I-003/80/2006. Informed consent was obtained from every patient entered into the study.

### Measurements

Exhaled nitric oxide (F_ENO_) was measured in all of the asthma patients and healthy subjects by the chemiluminescence technique using a Sievers 280i NO Analyzer (Boulder, Colorado, USA). The measurements were performed at an expiratory flow of 50 ml/s [12]. The duration of exhalation had to be at least 6 seconds to produce a stable NO level for 3 seconds. All subjects had three recorded F_ENO _measurements. Repeated measurements were performed until the 3 values agreed within 10% of the mean. The mean value of the three measurements was recorded as the final F_ENO _level.

The baseline spirometry was performed using a MasterScreen Pneumo PC spirometer (Jaeger, Hoechberg, Germany). Spirometry was performed according to ATS standards [13]. FEV_1 _(forced expiratory volume in one second) was evaluated. Before the examination the patients did not take any medications that could change spirometry results. The highest value from three technically satisfactory attempts was attached.

A non-specific bronchial provocation test with histamine (BPT) was carried out according to the method described by Ryan et al [14]. Provocation was performed using a De Vilbiss nebuliser 646 (Viasys Healthcare GmbH, Hoechberg, Germany) at an air pressure of 0.15 MPa linked to a Rosenthal-French dosimeter (Baltimore, USA). The results were presented as PC_20 _FEV_1 _– concentration of histamine, which causes a decrease in FEV_1 _of exactly 20% in comparison to initial values.

An exercise test was performed on a bicycle ergometer for 9 minutes with a fixed work load adjusted to increase the heart rate to 85% of the maximum predicted for the age of each patient [15]. Basic spirometric parameters were recorded before, and immediately after, the exercise test, and 1, 5, 10, 15, 20, and 60 minutes after completion of exercise. Those patients whose maximum decrease in FEV_1 _was greater than 15% were considered to have EIB.

EBC was collected by using a condensing chamber (EcoScreen; Erich Jaeger GmbH, Hoechberg, Germany). Exhaled air entered and left the chamber through one-way valves and the inlet and outlet, thus keeping the chamber closed. A low temperature inside the condensing chamber throughout the collection time produced a cooling down sample. The temperature of collection was around 0°C [10,16]. Exhaled breath collections were performed before, 10, 30, 60 minutes, 6 and 24 hours after the exercise challenge test. Patients were instructed to breathe tidally for 10 minutes with nose clip. The respiratory rate ranged from 15–20 breaths/minute. Patients were asked to swallow their saliva periodically and to temporalily discontinue collection if they needed to cough. At the end of collection 1.5- to 3.5 ml aliquots of condensate were transferred to Eppendorf tubes and immediately frozen. Samples were stored at -80°C [17].

Serum total IgE concentrations was measured using ImmunoCAP™ Technology (Pharmacia Diagnostics, Uppsala, Sweden). Blood eosinophil count was measured using a hematologic analyzer (Coulter Electronics GmbH, Miami, Florida, USA). Concentrations of ET-1 in EBC were determined using enzyme immunoassay kits for quantitative determination (ET-1 – Biomedica Gruppe, Vienna, Austria). Detection limit (0 fmol/ml + 3 SD): 0.02 fmol/ml.

### Analysis

Statistical significance was analyzed by using analysis of variance (ANOVA). All values were expressed as means ± SD; p values < 0.05 were considered significant. PC_20 _values were logarithmically transformed for analysis. The relationship between studied parameters was assayed by correlation. Pearson's linear correlation coefficient was used.

## Results

Characteristics of patients and healthy volunteers are presented in table 1. Table 1.

**Table 1:** Characteristics of study subjects and healthy volunteers

Characteristics.	Dimension.	Patients with EIB.	Patients without EIB.	Differences between asthma patients with and without EIB.	Healthy volunteers.
Number of patients		11	8		7
Sex	F/M	7/4	5/3		4/3
Age	Years	27.36 ± 7.50	31.63 ± 5.40	p = 0.19	28.40 ± 4.90
Duration of symptoms	Years	3.70 ± 4.63	4.12 ± 3.54	p = 0.32	
Baseline FEV_1_	% predicted	95.63 ± 18.54	92.25 ± 8.61	p = 0.63	106.85 ± 9.73
Maximum decrease in FEV_1 _after exercise	%	25.8 ± 13.5	3.6 ± 1.9	p = 0.0003	0.71 ± 3.2*^+^
Log PC20hist FEV_1_	mg/ml	-0.59 ± 1.16	-0.05 ± 0.55	p = 0.24	
Blood eosinophil count	cells/mm^3^	239 ± 138	157 ± 66	p = 0.14	51 ± 26*^+^
Serum total IgE	kU/L	358 ± 322	171 ± 69	p = 0.13	65 ± 31*^+^
Baseline F_ENO_	ppB	98.90 ± 55.37	66.62 ± 23.05	p = 0.21	18.00 ± 5.59*^+^
Baseline ET-1	fmol/ml	0.88 ± 0.24	0.74 ± 0.25	p = 0.29	0.59 ± 0.18*

Data are presented as mean ± SD

FEV_1 _– forced expiratory volume in one second

PC20histamine FEV_1 _– provocative concentration of histamine that caused a 20% fall in FEV_1_

* Values significantly different from patients with EIB, p < 0.05

^+ ^Values significantly different from patients without EIB, p < 0.05

In the studied group of asthmatics, 11 patients had a positive and 8 had a negative exercise test. In none of the healthy volunteers were spirometric indices worse after exercise.

Blood eosinophilia, baseline F_ENO _and total IgE were statistically significantly higher in both groups of asthmatics compared with healthy volunteers. In the group of patients with positive exercise tests compared to patients without EIB we observed higher blood eosinophil counts, serum levels of total IgE and baseline F_ENO_, but these differences were not statistically significant.

We revealed statistically significant higher levels of ET-1 in EBC in all studied asthmatic patients compared with healthy controls (0.83 fmol/ml ± 0.24 vs. 0.59 ± 0.18, p = 0.02). There was no statistically significant difference between the concentration of ET-1 in EBC before exercise in asthmatics patients with EIB in comparison to asthmatics without EIB (0.88 fmol/ml ± 0.24 vs. 0.74 ± 0.25, p = 0.29). In the group of healthy volunteers we observed the lowest levels of ET-1 in EBC, but this difference was statistically significant only comparing with asthmatics with EIB (asthma with EIB vs. healthy volunteers: 0.59 fmol/ml ± 0.18, p = 0.018; asthma without EIB vs. healthy volunteers: p = 0.13).

**Figure 1:**
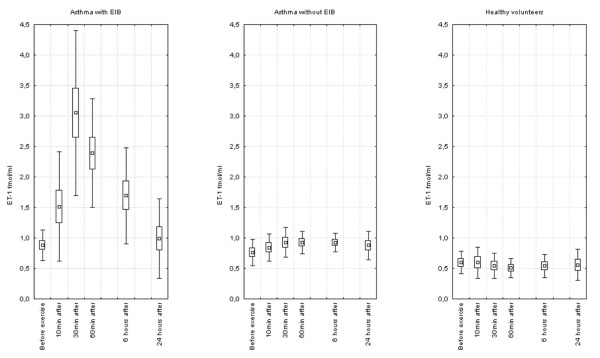
Concentrations of ET-1 in EBC at rest, and subsequent changes which were observed during the 24 hours after exercise test in groups of patients with asthma and healthy volunteers.

A statistically significant increase in the concentration of ET-1 in asthmatic patients with EIB was observed (10 min after exercise: 1.64 fmol/ml ± 1.27, 30 min after exercise: 2.91 fmol/ml ± 1.18, 60 min after exercise: 2.38 fmol/ml ± 0.89, 6 hours after exercise: 1.69 fmol/ml ± 0.78,) (p < 0.001). The concentration of ET-1 had returned to the initial level 24 hours after exercise (0.98 fmol/ml ± 0.65). No effects of the exercise test on changes in the concentrations of ET-1 in EBC in either asthmatic patients without EIB or healthy volunteers were observed. Figure 1.

There were no statistically significant correlations between the baseline concentrations of ET-1 in EBC and other studied parameters in either group of asthmatic patients or the healthy volunteers and the decrease in FEV_1 _after exercise in asthmatics with EIB.

**Figure 2:**
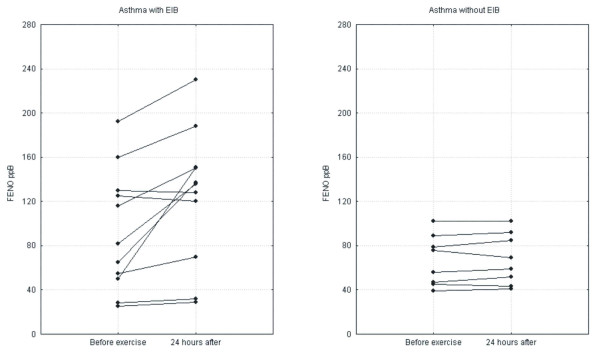
Changes in F_ENO _24 hours after exercise in the groups of asthmatic patients.

**Figure 3:**
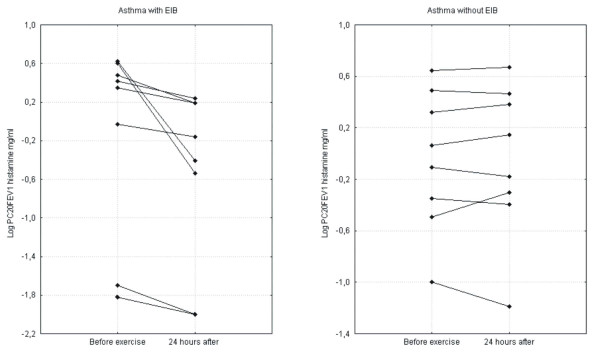
Changes in BHR to histamine expressed as the histamine logPC_20 _24 hours after exercise in the groups of asthmatic patients.

24 hours after the exercise test, in the group of asthmatics with EIB, a statistically significant increase in F_ENO _(before exercise: 98.90 ppB ± 55.37; 24 hours after exercise: 119.18 ± 64.39; p = 0.034) and BHR to histamine (log PC_20_FEV_1 _before exercise: -0.59 mg/ml ± 1.16; 24 hours after exercise: -0.95 ± 1.03; p = 0.0009) was revealed. Figure 2, Figure 3. Such changes were not observed in the group of asthmatic patients without EIB (F_ENO _before exercise: 66.62 ppB ± 23.05; 24 hours after exercise: 67.87 ± 23.03; p = 0.25; log PC_20_FEV_1 _before exercise: -0.053 mg/ml ± 0.55; 24 hours after exercise: -0.0511.62 ± 0.59; p = 0.99). In neither group of asthmatics did we detect significant changes in FEV_1 _24 hours after exercise.

**Figure 4:**
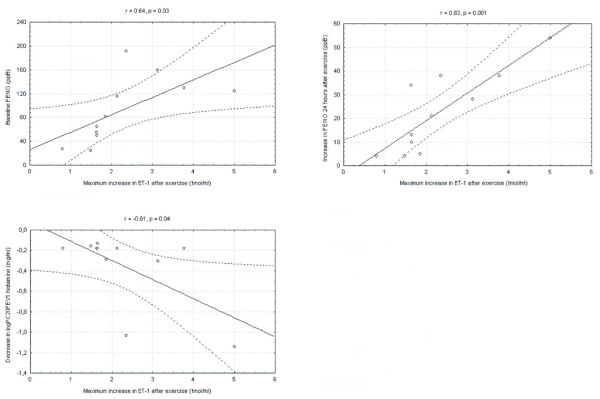
Correlations between the maximum increase in ET-1 in EBC and either baseline F_ENO _or changes in F_ENO _and BHR to histamine 24 hours after exercise in the group of asthmatic patients with EIB.

A statistically significant correlation between the maximum increase in ET-1 concentrations in EBC after exercise and either baseline F_ENO _(r = 0.64, p = 0.03) and the increase in F_ENO _(r = 0.83, p = 0.001) or the increase of BHR (expressed as decrease in logPC_20_FEV_1_; r = -0.61, p = 0.04) 24 hours after exercise in the groups of asthmatics with EIB was revealed. Figure 4.

## Discussion

The findings related to the participation of inflammatory mediators in either the maintenance or induction of bronchoconstriction provoked by exercise are conflicting. However, many reports demonstrate that EIB could have an inflammatory basis [18]. There is no information concerning the late consequences of many years of respiratory tract stimulation by exercise-induced bronchoconstriction. Epithelial remodeling was previously described in ski athletes who developed asthma symptoms and bronchial hyperreactivity after repeated bouts of exercise in cold dry air [19].

In our previous studies we revealed that bronchoconstriction following an exercise challenge in asthmatics leads to pulmonary endothelium changes, which in turn activate and release mediators (such as endothelin-1), causing the increase of airway inflammation and, as a consequence, airway remodeling [8].

In human airways, immunoreactive ET-1 is located principally in the bronchial epithelium and its expression at this site is increased in asthma [7,20]. The study of Black et al has indicated that airway epithelium could produce and release endothelin [21]. Elevated BAL fluid levels of ET-1 have been observed in asthmatics when compared with normal control subjects – the highest levels being found in patients with the most severe disease [22,23]. Except for human bronchial epithelial cells [24], ET-1 is produced by vascular endothelial cells [25], and inflammatory cells such as macrophages [26], mast cells [27], as well as alveolar epithelial cells [28].

Many interactions between ET-1 and other cytokines essential in asthma have been described. Xu et al have demonstrated that tumor necrosis factor-α (TNF-α) – an important mediator in initiating airway inflammation by activating the secretion of cytokines from a variety of cells – induces secretion of ET-1 from cultured bronchial smooth muscle cells [29]. ET-1 can induce expression of granulocyte-macrophage colony-stimulating factor (GM-CSF) in human lung fibroblasts and, through this, could directly affect recruitment of eosinophils in the airways [29]. Cunningham et al have reported that ET-1 stimulates monocytes to release GM-CSF, IL-6, IL-8, IL-1, TNFα, and TGF-α [30]. ET-1 induces the proliferation and fibrosis of airway smooth muscle cells. The interaction between ET-1 and other cytokines which are growth factors for bronchial subepithelial myofibroblasts may play a key role in remodeling in asthmatic patients, which is the consequence of repeated episodes of epithelial damage and repair in asthmatic inflammation [31]. In response to mechanical stresses similar to those occuring in vivo during airway constriction, increases in soluble levels of ET-1 and TGF-β1 have been observed [32].

ET-1 may contribute significantly to the remodeling of the airway by slowing epithelial cell migration as well as increasing proliferation of airway fibroblasts and smooth muscle cells. In turn, this process results in delayed repair and enhanced fibroblast activation and remodeling. The damage of asthmatic airways by enviromental agents and allergens may be additionally increased by slower repair mechanisms in which ET-1 may be involved [33].

A number of studies have reported increased BAL fluid ET-1 levels in asthma patients, suggesting that this peptide may contribute to the elevated resting bronchomotor tone in this disease [23]. However, Makker et al do not support the hypothesis that ET-1 is involved in the bronchoconstrictor response induced in vivo by hyperosmolar saline [34]. The endobronchial hypertonic saline challenge does not completely reflect changes occurring in airways during and after postexercise bronchoconstriction, and the authors of this study could perform the determinations only few minutes after the application of hypertonic saline. Also Redington et al do not support the hypothesis that allergen exposure in asthma results in immediate release of endothelin. However, release at later time-points, and a role for endothelin in late-phase bronchoconstriction, are not excluded by the authors because the levels of ET-1 in BAL fluid were measured only 10 minutes after the endobronchial allergen challenge [35].

The aim of the present study was the assessment of the changes of ET-1 levels in EBC during the first 24 hours after postexercise bronchoconstriction. Exhaled breath condensate, collecting by cooling exhaled air, is a noninvasive, easily performed, effort independent and rapid method for obtaining samples from the lower respiratory tract. EBC contains a large number of mediators including leukotrienes, prostaglandins, adenosine, and 8-isoprostane. Concentrations of these mediators have proved to be a useful noninvasive method for the assessment and monitoring of airway inflammation. EBC collection is well tolerated by patients, can be performed repeatedly at short intervals, and does not alter airway function or inflammation [16]. Therefore this method makes possible the observation of the dynamic of changes in ET-1 levels. The monitoring of ET-1 levels 24 hours after exercise using noninvasive methods and correlations of obtained results with other markers of airway inflammation have made possible the assessment of the participation of this mediator not only in acute bronchoconstriction, but first of all in the increase of airway inflammation during postexercise bronchoconstriction.

In the previous studies elevated levels of other inflammatory mediators (such as adenosine and Cys-LT) in EBC were observed in asthmatics with EIB. Csoma et al revealed pronounced increase in adenosine level in EBC during EIB in asthmatic patients and this increase was related to the degree of bronchospasm [36]. Carraro et al observed higher baseline EBC Cys-LT in asthmatic children with EIB and these values correlated with the decrease in FEV1 after exercise [37].

In the present study, the highest baseline concentration of ET-1 was observed in asthmatic patients with postexercise bronchoconstriction. However, the statistically significant changes in the levels of this parameter were demonstrated only in comparison with the group of healthy volunteers. This minute difference could be the consequence of the fact, that the study was performed in the group of mild asthmatics with short time-course of the disease. Only in group of patients with EIB was a statistically significant increase in ET-1 levels in EBC collected between 10 minutes and 6 hours after exercise observed. The maximum increase of ET-1 was correlated with baseline exhaled nitric oxide levels – which has become a more and more appreciable criterium for the evaluation of airway inflammation [38] – as well as with the increase of F_ENO _and bronchial hyperreactivity to histamine, 24 hours after exercise.

## Conclusion

This study was performed to clarify the possible role of ET-1 in the pathogenesis of EIB, particular in the inflammatory basis of this condition and the remodeling of the airways. We show that, as a result of intensive exercise leading to bronchoconstriction, the increase in ET-1 level in EBC occurs. Based on these findings, it is considered that the release of endothelin-1 through interactions with other cytokines and the influence on many airway cells essential in asthma, may contribute to the exacerbation of asthmatic inflammation in the airways and bronchial hyperreactivity after exercise. This process is not presented in asthmatics, in whom post-exercise bronchoconstriction does not occur. Prevention of post-exercise bronchoconstriction by proper anti-inflammatory treatment may play a crucial role in limiting the effect of EIB on airway inflammation as well as remodeling in asthmatic patients.

## Competing interests

The authors declare that they have no competing interests in the publication of the manuscript. This work was supported by research grant No 3-35523P from the Medical University of Bialystok, Poland.

## Authors' contributions

ZZ conceived the trial, participated in its design, study procedures, interpretation of results, performed the statistical analysis and helped to draft the manuscript. RS participated in the study procedures, laboratory tests and helped to draft the manuscript. MMT participated in the study procedures and helped to draft the manuscript. AB-L participated in study design, interpretation of results and helped to draft the manuscript. All of the authors read and approved the final manuscript.
